# Integrating “One Health” Concepts in the Design of Sustainable Systems for Environmental Use

**DOI:** 10.3390/toxics11030280

**Published:** 2023-03-19

**Authors:** Mark S. Johnson, Valerie H. Adams

**Affiliations:** U.S. Defense Centers for Public Health—Aberdeen, Toxicology Directorate, 8252 Blackhawk Road, Aberdeen Proving Ground, MD 21010-5403, USA

**Keywords:** one health, green chemistry, matrixed approach, toxicity, environmental fate and transport, environmental release, ecotoxicity

## Abstract

Ensuring for the national defense requires the use of substances such as energetics, propellants, pyrotechnics, and other materials in environmental applications. Systems that use these materials do so in testing and training environments and must be used in an environmentally sustained manner to ensure success during actual kinetic defensive operations. Environmental and occupational health assessments require a weighted evaluation of toxicity, bioaccumulation, persistence, and environmental fate and transport considerations for each substance in the formulation to include potential combustion products. Data that support these criteria need to be collected in a phased and matrixed approach and considered iteratively as technology advances. Further, these criteria are often considered as disparate and separate; hence, comparing favorable aspects of one may or may not offset detrimental data from another. Here, we describe an approach to the phased collection of environmental, safety, and occupational health (ESOH) information for new systems and substances and provide recommendations for evaluating such data streams in making decisions for use and for evaluating alternatives.

## 1. Introduction

Approaches that consider the environment, safety, and occupational health (ESOH) in the development and use of new substances are prevalent in many industries due to unexpected consequences when only function is considered. Often termed “green chemistry”, many entities have developed methods, tools, and systems for considering ESOH along with other life cycle costs in the evaluation of new substances and potential alternatives [1,2,3].

Unexpected costs associated with production and use of some substances in the military has halted training activities, resulted in increased production costs and delays, and affected the health of warfighters and workers exposed to these materials. In many cases, substitutions are not available, and means to reduce exposures are the only options. Hazardous wastes that accumulate at production facilities must be disposed in costly hazardous-waste-permitted landfills. Many substances were developed and used with only a minimal investment in the necessary toxicology data needed to allow program managers to make informed and balanced decisions. Hence, accurate life cycle costs were rarely captured or debated. Examples include the use of the oxidizer, ammonium perchlorate, that was considered acceptable on the basis of its relatively low acute toxicity. However, low-level, long-term exposures were found to affect thyroid hormone production through inhibition of the sodium iodide symporter affecting iodide uptake [4,5]. Moreover, the perchlorate anion is very water soluble and was found to infiltrate soils at production and testing facilities and subsequently found in some ground water resources. This has become an issue of national importance in the United States, and research into alternatives has begun. Other examples include the explosive, 1,3,5-hexahydro-1,3,5-trinitrate (RDX), that has been found in ground water resources and has been found to migrate off military installations, affecting drinking water sources [6].

Development of toxicology data must not be a costly endeavor and should be conducted in a phased manner coincident with the level of investment devoted to new compound development and weapon system design. The use of the phased approach to gathering these data where ESOH testing is accomplished alongside research, development, testing, and evaluation can provide results that can maintain mission requirements and keep projects and costs on schedule.

The “One Health” concept considers all potential linkages of exposure and effects to include those that occur from environmental releases to non-human entities and how that may subsequently affect public health and other environmental processes. It includes fate and transport of parent compounds, combustion, environmental breakdown of products, and how exposure may affect ecosystem processes and human health. This vision is consistent with green chemistry approaches and full life cycle assessment.

## 2. ESOH Data Considerations

Specific data are needed to evaluate hazard and for specific tools to be used to ensure safe and sustained use. Although there are policies and requirements which require program managers for weapon systems and platforms to consider and integrate ESOH concerns into full life-cycle considerations, rarely are specific toxicology data requirements specified. Moreover, there is no guidance how data are evaluated relative to different evidence streams (i.e., how human toxicity data are compared with bioaccumulation information, environmental persistence, and environmental fate and transport).

Clearly, toxicity data from focused studies or tests are needed to determine hazard from exposure with regard to adverse effects, both for humans (e.g., workers) and environmental receptors (e.g., aquatic organisms); however, there are other aspects of environmental use that must also be considered. Recently, the concern for per- and poly-fluorinated alkyl substance (PFAS) contamination has heightened the consideration of environmental persistence and bioaccumulation. Many of these substances are not acutely toxic and require very significant exposure to result in adverse effects—instead, they typically elicit effects from low-level continuous exposures that bioaccumulate within the organism. Past experience with halogenated organic compounds (e.g., polychlorinated and polybrominated biphenyls) has shown environmental persistence also to be an issue that must be considered in hazard assessment. Other factors, including environmental fate (products of decomposition or environmental transformation) and transport (e.g., propensity to reach groundwater resources) should also be investigated. All these factors must be considered as separate vectors, as they are evaluated and measured using different approaches.

Military research for new weapon systems can involve more than new compound development. An understanding of mixtures to include new formulations and additives and engineering of systems is critical. Optimally, ESOH data streams are interpreted and provided to decision makers within acquisition programs to inform implementation of new and emerging technologies. The responsible entity of this new technology becomes the Program Manager, who must understand the full life cycle costs associated with implementation. It is the Program Manager who has the responsibility to consider full life cycle costs to include environmental sustainability. Each system requires a Programmatic Environmental Safety and Health Evaluation (PESHE), a Life Cycle Environmental Assessment, and other documents to assist with those decisions. Although not required by regulation, several guidance documents exist to assist with data collection and assessment of new substances for environmental use [7,8,9].

Acquisition programs require many levels of oversight and areas of consideration. Program Managers are best served by accepting new technologies with sufficient ESOH information and when recommendations are provided. In Army programs, a Toxicity Assessment is a technical foundation that contains toxicity information on substances used in new systems where there is a potential for exposure either in manufacturing, use, or demilitarization. If the potential for release to the environment is present, environmental fate and transport and ecotoxicity are evaluated. Typically, Toxicity Assessments can serve as a resource for other broader evaluations (e.g., the PESHE) that are requirements for the program.

## 3. ESOH Data Requirements

As previously mentioned, few specific data requirements are addressed for many military programs. Because of previous experience, approaches to the acquisition of chemical/physical property and toxicity data have been developed. Many follow and/or build on simple green chemistry procedures and are phased with the level of investment dedicated to program research [6,9]. Some studies, because of the relative effort involved, are best handled in the acquisition stages.

### 3.1. Approaches

Some regulatory agencies have specific requirements for chemicals based upon annual production volume and other criteria (e.g., USEPA and EU (e.g., REACH)). Many approaches used in green chemistry applications begin with an iterative interaction between developer and the ESOH professional where ESOH criteria (e.g., toxicology and chemical/physical properties) are considered equivalently alongside other performance criteria. Initially, data are collected that are commensurate with the level of investment devoted to the research program. This typically means a high reliance on screening assays (with high false positive rates and low false negatives) that are inexpensive and high in uncertainty yet provide value for relative comparisons. 

#### 3.1.1. Phased Approach

**Figure 1:**
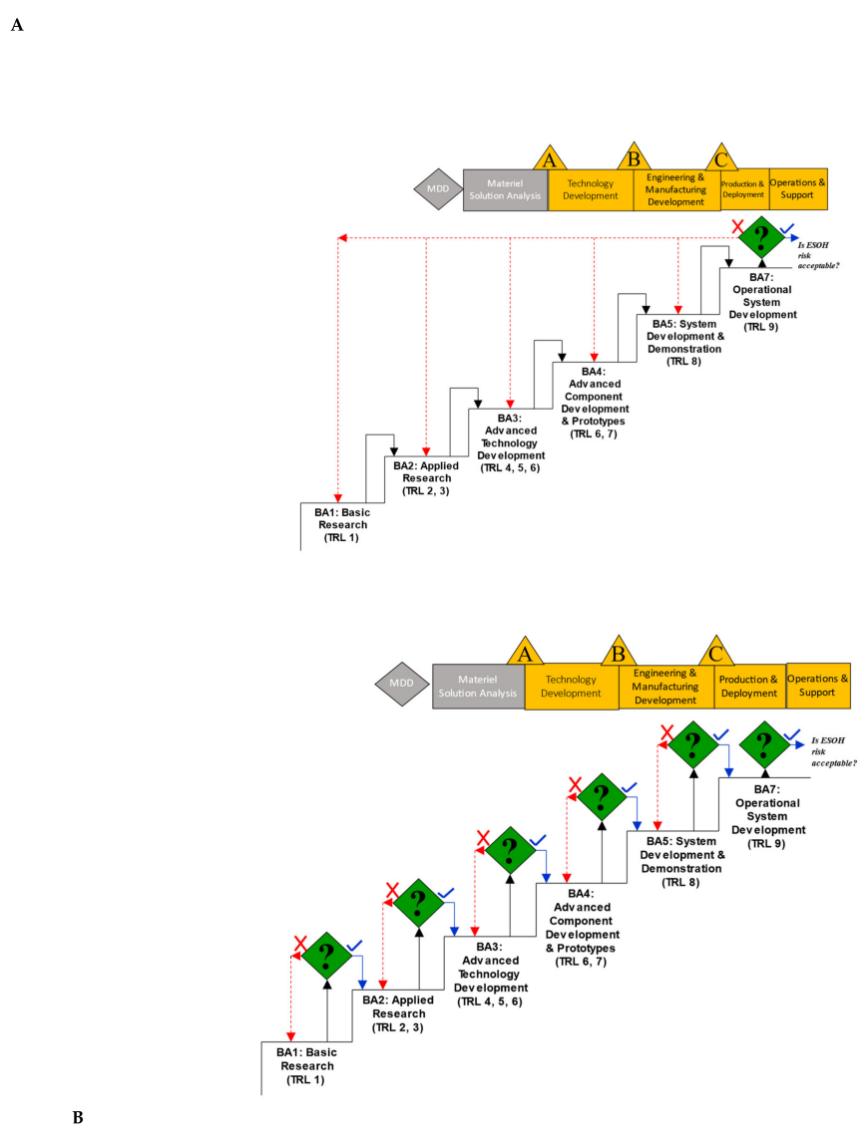
Unacceptable (**A**) and optimal (**B**) conceptual approaches to obtaining and assessing environmental, safety, and occupational health information with increasing technology readiness levels within research and acquisition programs in system development. BA—budget activity, TRL—technology readiness level.

As mentioned, phased approaches are recommended for obtaining data for use in ESOH assessment. Optimally, specific ESOH data are collected alongside other performance criteria and considered as such when making decisions to continue research and development and ultimately implementation. The three methods discussed here are specific to military applications; however, others have been developed for other applications, such as pharmaceuticals and other industrial materials [7,8,9,10]. Logically, specific data are required at specific levels of research or technology readiness levels (TRLs; Figure 1). As suggested, this process begins early in conceptualization and builds on subsequent information through acquisition.

Stakeholders vary depending on the TRL and Budget Activity (BA). Figure 1 Initially, principal investigators researching new substances (and their funding agents) are engaged in developing new substances first by using models and computer simulations. Often, these substances have not been synthesized; however, quantum mechanical models can predict chemical properties (e.g., water solubility, vapor pressure, and octanol–water partition coefficient (K_ow_)) that provide estimates for determining bioaccessibility and environmental fate and transport (see Table 1) [8,9]. In addition, toxicity predictions can be accomplished through in silico computer models once the structure is known. This occurs in the Conception Stage (TRL-1 [8,9]).

When the modelled substance is synthesized, only gram quantities are made (TRL2-3, Synthesis stage). At this stage, limited in vitro tests for mutagenicity/genotoxicity, hepatotoxicity, and other endpoints predicted by in silico or read-across applications can be conducted using new approach methods (NAMs) [8,9].

Following the Synthesis Stage, researchers focus on the synthesis scale-up and formulation modifications to improve performance. It is in this Testing or Demonstration stage where exposures to people and the environment can occur; therefore, laboratory animal acute and repeated dose studies are recommended. [8,9]. Additional aquatic ecotoxicity data are needed if discharge into wastewater or the environment is expected.

At subsequent stages, ESOH data requirements include industrial hygiene considerations where occupational health criteria are needed. Here, subchronic controlled laboratory rodent data are required and the technology advances into program management and moves from RDT&E.

Decision authorities often do not possess the abilities to decipher and evaluate the data collected during this process. Here, ESOH professionals who have backgrounds in toxicology fate and transport are required. Each test or assay provides specific data that can be initially used in categorization (e.g., Global Harmonized System (GHS) categories) [11]. Data can also be used for comparing the relative toxicity of substances tested within each assay so that substances can be ranked per endpoint and for interpretations of data collected from many in vitro tests when target tissue concentrations are not known.

Optimally, each subsequent data collection event (e.g., bioassay) would build on knowledge collected from previous tests, reducing uncertainty at each step. As the materials are tested and determined to be efficacious in their intended application, more detailed toxicity data are collected to better ensure health of the Warfighter, worker, and sustainable use in the environment.

#### 3.1.2. Evolving Science and New Tools

An integral part of tiered or phased approaches initially assumes a relatively low level of effort and cost that increases with the level of success and investment in the development of a new substance to be used in a new system. Inversely proportional to the low initial level of investment is the level of uncertainty of the information that is obtained. Conceptually, as greater success is realized in research and development, greater resources are devoted to understanding the toxicology of new substances and formulations, and the uncertainty associated with potential adverse ESOH outcomes is proportionally reduced.

The science and tools now available for toxicology and fate and transport investigations has broadened and continue to advance. The advent of high-throughput screening and the U.S. National Academy of Sciences publication of Toxicity Testing in the 21st Century has become a guiding force in advancing the use of new biomedical science in toxicity evaluation [12]. However, many of these tools are lacking in terms of refinement (i.e., aid in the understanding of variation in results and the uncertainty associated with the outcome.) In some cases, additional research is needed to ascertain the level of precision and accuracy in test results.

However, some of these new bioassays and tools clearly have current applicability at the synthesis and testing stages (i.e., TRL 1–3), where it is acceptable to have relatively high levels of uncertainty. Computational (in silico) chemistry models can be used during conception stages that require only knowledge of chemical structure to provide toxicity and chemical/physical property estimates that can be used in predicting fate and transport. Read-across methods, using chemicals with toxicity data that have similar chemical structures, can be used to infer relative toxicity of new molecules that are structurally similar. New in vitro methods can be used in early synthesis stages alongside similar substances (or replacements) for relative comparisons where only gram quantities exist. The use of appropriate positive and negative controls are informative and provide confidence in the results. Although in vitro results often neglect important exposure factors that define target dose to tissue, the results can be used in a relative comparison to replacements. Note that it is not yet advisable to rely on these methods as full toxicity testing replacements for standard test methods used at the production/use phase (TRL 5–7) when a greater level of certainty may be needed. However, information gained can be used to build on subsequent stages and save time and resources on more focused in vivo tests when greater certainty is required.

#### 3.1.3. Research vs. Testing

While basic research often generates more questions than answers, ESOH testing requires applied, end-point driven experiments that generate specific information for risk assessment. Resources invested in gathering ESOH data (e.g., toxicology tests) should produce information that can be used to make risk-based decisions. Therefore, care must be taken to conduct testing with a clear understanding of how various data outcomes would be used a priori to make decisions. If it is determined that these data would not allow for an ESOH-based decision to be made, then the test should not be conducted.

Additionally, it is important that qualified institutions conduct these tests and that subject matter experts be consulted beforehand to inform study design and selection. Often decisions are made considering positive (i.e., statistically significant) results, and negative data rarely have weight when any positive relationships exist. Simply put, positive results (even false positive) are rarely discounted and are perpetuated, while negative (no effect) data are often dismissed. However, when assays with high false positive yet low false negative rates are used, they have utility as screens (and weight-of-evidence approaches) for some toxic endpoints (e.g., Ames assay and mutagenicity).

### 3.2. Utility of Flow Charts

With advancing science occurring at a fast pace, NAMs may be used to replace older established animal tests. Additionally, society is increasingly demanding reductions in the use of in vivo studies (laboratory animals) for ethical and financial reasons. Therefore, a question-based matrix or flow chart can be used to help guide additional testing, hence, ESOH concerns that include human health and the environment. Such a paradigm can be used throughout the process from initial to final development stages. Time to complete these requirements will vary upon the outcomes of the data collected at earlier stages, pathways of exposure, and complexity of the system.

It is important to note that ESOH concerns are broad and complex. These questions are not new and few comprehensive approaches for developing a question-based key exist. Toxicity depends upon exposure potential and exposure depends on system design, manufacture, use, and nature of environmental release (e.g., waste water discharge, incomplete high order detonation, low-order detonation or dud frequency, etc.). The value in such an approach is that it informs and focuses testing and allows the ESOH professional to use whatever tools are available to address the likelihood of the impact. The drawback is that uncertainty can be complex and multi-vectored, and the most appropriate assay or tool may not be available (or considered only within a specific context) to address the specific question. The level and direction of uncertainty should be considered at each developmental stage.

**Figure 2:**
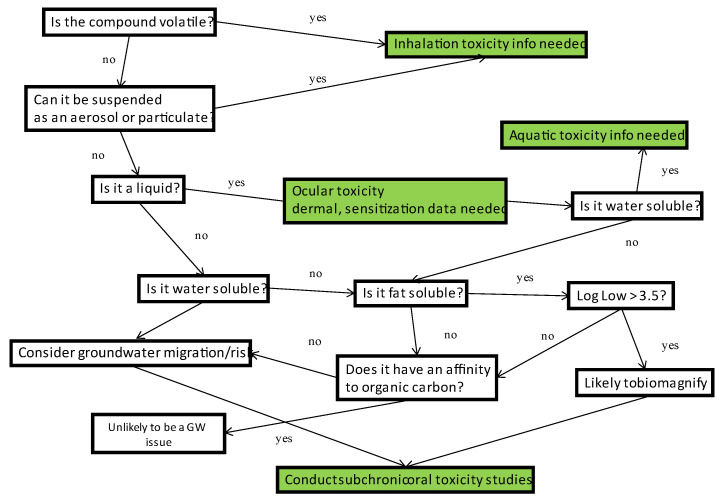
Example flow chart using chemical/physical properties to infer exposure pathways and toxicity data needed to address risk potential. Green boxes indicate data/studies required.

Addressing exposure can initially be accomplished through an analysis of the chemical’s physical properties (Table 2). For example, if the vapor pressure is relatively high, then exposure can be assumed to occur via the inhalation pathway, and inhalation toxicity data would be needed. If the new compound has high water solubility, then environmental releases may be assumed to reach ground water or move through surface/sheet water run-off. The former would require oral toxicity data for human health applications, and the latter would require aquatic ecotoxicity information. An example of a flow chart is provided in Figure 2.

### 3.3. Reproduction and Developmental Effects

Recently, many countries have changed or are changing policies associated with the role of females in military service. In many circumstances, females of child-bearing age are now on the front lines of military service where first-hand exposures to obscurants, combustion products from weapon systems, and exposures to other materials (e.g., smoke mixtures, pyrolysis products from fire extinguishing agents, etc.) have the potential to affect normal fetal development at a period potentially before Warfighters realize they are pregnant. This is particularly important in exposure scenarios, such as submarine environments and other confined spaces. Very few chemicals have developmental toxicity data to enable the determination of risk from exposure. Often, adverse developmental outcomes originate from substances that have a genotoxic mode of action—that is, exposure at early embryonic stages to genotoxic chemicals may affect genomic integrity leading to developmental abnormalities. However, environmental factors can also cause adverse developmental outcomes.

Adverse reproductive effects (i.e., substances that adversely affect the normal operation of the reproduction system) can occur from a variety of mechanisms. Compounds that act to impede or accentuate the endocrine system or those that are directly toxic to germ cells or supportive tissues (e.g., Sertoli cells) can reduce fertility.

Assessing the potential of a compound to cause adverse reproductive or developmental effects is relatively complex and requires integration into other existing data plans. Typically, it is reasonable to investigate potential reproductive effects when there is a potential for exposure, when there is evidence of endocrine disruption, and/or when there is evidence that the reproductive organs are affected from repeated exposures using animals (e.g., a subchronic rodent bioassay).

In the past, failure to address the impact of reproductive effects for substances that are released to the environment has caused marked changes in the ecosystem and has led to much legislation (e.g., DDT effects on predatory birds, PCBs, etc.). If effects are observed either from histological change to reproductive organs as part of a repeated dose in vivo study or if there are in vitro data that suggest endocrine activity of the new molecule, then a phased approach to investigating reproductive effects are needed. Examples of tiered approaches for testing the potential for endocrine disruption can be found at https://www.epa.gov/endocrine-disruption (accessed 15 March 2023).

**Figure 3:**
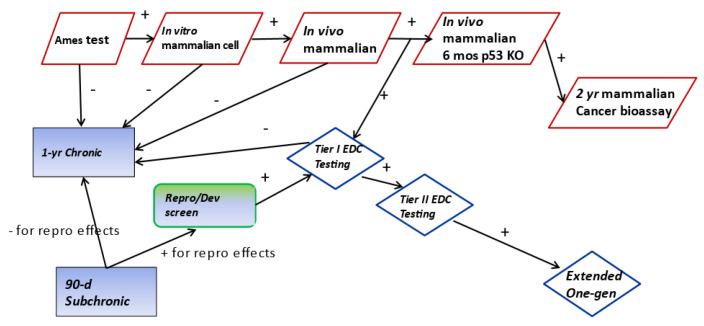
Example flow chart with appropriate bioassay to be used in a phased manner to address the potential for genotoxicity. Boxes with blue background are used as part of other flow charts to understand potential for non-genotoxic events.

Understanding pathway-specific toxicity potential for systemic genotoxic effects is important in predicting some specific toxic outcomes. Compounds that cause changes to the genome can conceptually lead to the development of cancer or issues for the developing fetus (developmental effects). Hence, other flow charts can be developed to help characterize genotoxicity (Figure 3). If the results of these tests are positive, it is also advisable that an in vivo testing paradigm that is designed to consider developmental effects be used.

### 3.4. Balancing Human Health, Environmental Toxicity, Persistence, and Fate and Transport

**Figure 4:**
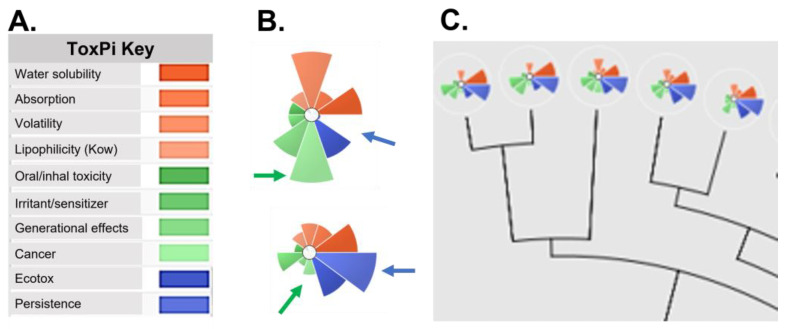
ToxPi footprint of two constituents. (**A**) The endpoints of interest are sorted into 10 categories that represent human, ecological, and environmental properties of concern. (**B**) Two chemicals evaluated with ToxPi that demonstrate different patterns of toxicity. Green arrows show high concern for cancer (top) compared with low concern (bottom). Conversely, the top compound has low concern for persistence, while the bottom compound is scored as persistent (blue arrows). (**C**) ToxPi also generates a dendrogram of the evaluated compounds so that similarly scored compounds are clustered.

Using a ‘cradle to grave’ paradigm, One Health encompasses not only adverse effects from occupational exposures during system deployment but the potential for long-term environmental and ecological toxicity hazards from the system release, storage, and disposal. System constituents of concern (SCOC) that are persistent, bioaccumulative, and toxic (PBT) or are persistent, mobile, and toxic (PMT) should be identified early in the hazard assessment and removed from formulations prior to system scale-up. Key physicochemical properties for assessing persistence, and chemical fate and transport include water solubility, K_OW_, and K_OC_. Of note, although in silico models are often used to predict these properties, experimental data are preferred, especially where there are few or no chemical replacements for the SCOC. Additionally, toxicity from breakdown products—either via combustion or environmental processes, such as hydrolysis and weathering—should be considered in the life cycle assessment. Organizing, ranking, and presenting these disparate toxicological categories to stakeholders in a meaningful and interpretable format is challenging. Data visualization using color-coded matrix tables and graphs is important for comparing the hazard ‘footprint’ of systems and SCOCs. One tool for visualizing data and facilitating chemical or system prioritization is the Toxicological Priority Index (ToxPi) [13]. ToxPi software is used to calculate a numerical value on the basis of ordinal data for an endpoint of interest. During the development of the ordinal data for inclusion in the ToxPi tool, the strategies for data analysis and transformation are documented—making the ToxPi process transparent and reproducible. Each number is represented as slice of a pie chart, with the length of the ray directly proportional to the hazard. The composite charts for systems and system constituents are then compared. An example of ToxPi data visualization is provided in Figure 4. 

### 3.5. Mixtures

Few new substances are used alone—they are often incorporated into formulations with other substances that work together to perform a function using a specific application process. All substances in the final formulation are evaluated initially separately according to the criteria previously mentioned. Occasionally, toxicity data or other chemical physical properties are not available to evaluate fate and transport and effects. Typically, if the formulation contains constituents that are less than 3% of the volume and are not substances that are persistent or bioaccumulative (e.g., metals or halogenated organics), they pose little hazard and can be considered insignificant. However, toxicity testing of the complete formulation can be helpful to consider if general concentration addition is accurate regarding toxicity. Occasionally, some substances in the formulation may enhance the toxicity of some of the constituents, typically through enhancing kinetics (e.g., through enhancing absorption), although the opposite can also occur. Complete formulation toxicity testing through in vitro or in vivo means can help address these uncertainties.

## 4. Discussion

### 4.1. Current and Evolving Regulatory Interests

The EU and USA have recently initiated legislation to comprehensively evaluate the potential ESOH effects of new compounds still in their conceptual stage and not in production. Specific military exemptions do exist; however, current sustainable use while training, safe manufacturing, and safe use by the warfighter suggests the collection and assessment of ESOH data to be a worthwhile endeavor with a high potential for a return on investment.

Many jurisdictions and corporations involved in chemical research and production realize that asking these questions early is advantageous and practical. However, they note that the abundance of chemicals in commercial production exceeds the capabilities to conduct thorough and complete vertebrate bioassays [12]. Therefore, an integrated approach that uses novel in silico and in vitro tools with conventional bioassays and read-across techniques can provide useful information for decision making. In vitro–in vivo extrapolation (IVIVE) is an area that is expanding in risk assessment and is particularly useful when comparing these human-equivalent values with human data and data extrapolated from controlled laboratory animal investigations. Regardless, such data are helpful in a weight-of-evidence context in using techniques such as read-across in helping to focus on in vivo studies and reduce the use of animals in toxicity studies.

Clearly, not every new substance must be tested but should be evaluated if there is significant exposure potential and hazard probability. The probability for hazard can be estimated and ranked, where those with the highest hazard rank can be evaluated first. Iterative discussions with system developers provides a framework for chemical down selection and sets criteria for choosing safer alternatives.

### 4.2. Case Studies and Cost Analysis

The process from development of new chemical structure to implementation to a weapon system is complicated and requires many qualification processes and steps. Few examples currently exist, as this process is relatively new. However, some can be discussed as follows.

M116, 117, 118 Simulators

The “whistle, bang, flash” simulators are used in training exercises to emulate combat simulations. The fuel for these simulators was previously potassium perchlorate, which was implicated in contributing significantly to groundwater contamination. The perchlorate anion was found not to be acutely toxic; however, it was subsequently found to interfere with the sodium iodide symporter in the thyroid affecting thyroid hormone production and thyroid cell growth, potentially leading to cancer [4]. Further, the perchlorate anion was found to be relatively stable and water soluble, resulting in groundwater contamination. The U.S. Army Environmental Quality Technology Program, Pollution Prevention Pillar devoted resources into the development of less toxic alternatives. Subsequently, systems were developed that used a black powder alternative (potassium, sulfur, and sodium nitrate) that was less expensive and maintained a similar level of expected performance.

M-18 Violet Smoke

M-18 colored smoke grenades are designed as signaling tools. Exposure when activated and thrown was considered to be minimal. However, subsequent use in urban situations has led to using these grenades as obscurants, where exposure to the warfighter was greatly increased, with adverse health consequences occurring as result. Much of the toxicity occurred from combustion of the fuel (e.g., hydrogen sulfide production); therefore, the fuel was reformulated replacing the sulfur with sugar. This created a change in the internal thermal dynamics, whereby dyes needed to be reformulated and the grenade re-engineered. Initial attempts using Solvent Violet 9 resulted in acute toxicity (mortality) in rats at the limit dose from a single 4 hr exposure. Equivalent performance results at no additional costs were obtained using a combination of Solvent Red and Solvent Blue dyes, which we subsequently found not to result in acute toxicity at the limit dose. This iterative exchange of information between developer and ESOH professional led to the development of a less toxic alternative with no increase in cost. Further development is underway.

Insensitive Munitions

New munitions are being developed to detonate only when intended, thereby reducing or eliminating sympathetic detonation—an event that can have catastrophic consequences. Here, program managers engaged ESOH professionals to understand the toxicity of the components, develop occupational exposure levels, and ascertain environmental impact from training, use, and production. Ecotoxicity data were gathered to obtain wastewater discharge permits at production locations. Environmental fate studies were accomplished to determine range sustainability from long-term use. Components were found to degrade under most environmental conditions into relatively inert substances.

### 4.3. Cost and Time Considerations

Cost and time to conduct studies to obtain ESOH information has always been an issue. Researchers and Program Managers often deal with limited resources, where these additional data requirements risk reducing available funding for research, development, and/or production, regardless of the great potential for return on investment. To date, funding devoted to ESOH data acquisition, compared with that devoted to research, has been typically less than 10% of the total program. Still, researchers and program managers need cost and schedule projections to plan accordingly.

Inherent in the collection of these data are resolutions to ultimate and proximate questions. This means these data can be used in a relative way to see whether the new technology will be less toxic than previous systems, or whether some data can be used to determine how much exposure would occur and what the risk would be. Examples of the latter include using inhalation toxicity data to develop an occupational exposure level (OEL) for industrial hygiene purposes—a requirement before manufacturing can occur. Another example includes using aquatic toxicity data to help determine whether release of manufacturing wastewater would be appropriate and legal. These data can also be used in sophisticated environmental soil and groundwater models to ensure that training and testing ranges can be used sustainably.

## 5. Conclusions

Understanding the environmental and occupational hazards from using weapon systems that result in release of substances to the environment is critical to ensure warfighter readiness (health and training) and sustainability in testing and training areas. Although the health and safety to the user and trainer is qualitatively understood, a matrixed approach to toxicology, ecotoxicology, and environmental fate and transport is required to make evidenced-based decisions for sustainable use. This matrixed approach requires consideration of disparate evidence streams (i.e., toxicology, persistence, bioaccumulation, and chemical/physical properties) along with conditions of use to characterize and manage ESOH risks. Presentation of information from these streams is recommended using “stop light” charts (developed from GHS toxicity categorization and ToxPI charts, although we recognize criteria (e.g., human toxicity, ecotoxicity, persistence, and bioaccumulation potential) may be offset.

This review provides the rationale and background for the collection of ESOH data to be used as additional performance criteria in the assessment of new molecules for weapon systems and platforms. It provides the context and considerations necessary for criteria to aid life cycle assessment. It puts forth a framework and method to integrate data into a preventive medicine/public health paradigm that will help to support the development of safe, sustainable energetics for use in the theater and at training ranges worldwide. As scientific advancements are made, this paradigm is flexible to include such advancements and provides the rationale for validation and refinement for new technologies as they are developed.

## Figures and Tables

**Table 1:** Chemical/physical property data needed to evaluate ESOH fate and transport.

Property/Attribute	Utility
Molecular mass (MW)	Determine dermal flux, understand excretion rates and pathways
Water solubility (mg/L)	Environmental fate and transport, exposure potential (e.g., gut absorption potential)
Fat solubility (octanol/water partition coefficient; log Kow)	Potential for gastrointestinal absorption and bioaccumulation/magnification between trophic levels
Vapor pressure (atm, torr)	Potential for inhalation exposures; environmental half-life (e.g., Henry’s Law)
Affinity to organic carbon (log Koc)	Fate and transport; soil sorption, potential to reach ground water from release.
Henry’s Law coefficient	Environmental half-life in surface water (often calculated)
Boiling point	Inhalation potential; environmental persistence
Melting point/ionization potential	Fate and transport

**Table 2:** Recommended minimum ESOH performance data by Budget Activity level.

Data	Standard Test Methods	Cost (USD K) ^i^	Time (Days)
**BA2**			
**Chemical/Physical Characterization**			
Material purity	Thermogravimetric analysis, Differential Scanning Calorimetry, Fourier Transform Infrared/Raman spectroscopy, Nuclear Magnetic Resonance, Gas Chromatography Mass Spectrometry	USD 25 K	Varies (approximately 30 d)
pH or pKa	OECD 122, OECD 112		
Vapor pressure	ASTM E1194-07 (withdrawn 2013); OECD 104; ARL-TR-6887, *New Micro*-*Method for Prediction of Vapor Pressure of Energetic Materials*, July 2014		
Water solubility	ASTM E1148-02 (withdrawn 2013, no replacement); OECD 105		
Hydrolysis *	ASTM E895, OECD 111, the EPA 712-C-08-012		
Octanol water partition coefficient (K_ow_)	ASTM E1147 (withdrawn 2013), OECD 123, OPPTS 830.77550		
Affinity to organic carbon (K_oc_) (calculated) *	OECD 121; Estimate Koc using Mackay function (Koc = 0.41- Kow)		
Henry’s Law constant (calculated) *	Calculated (H = (Vp * MW)/S, where Vp = vapor pressure (atm) at 25 C, MW = molecular weight (g/mol), S = solubility in water (mg/L)		
Dissolution rate *	ASTM E1624-94 (2008; withdrawn 2013). See ERDC’s method for munition dissolution, *Dissolution Kinetics of IMX 101 and IMX*-*104*, ERDC TR OP-F-15-1.		
**Human Health**			
Endocrine disruption—in vitro estrogen and steroidogenesis	OECD 455–457 (estrogen); 458 (androgens), 456 (thyroid); see Day et al. 2018.	USD 10 K	60
Mutagenicity, in vitro			
Bacterial reverse mutation (*Salmonella typhimurium*)	OECD 471	USD 6 K	35
Cytotoxicity, in vitro			
Mammalian cell viability assay (e.g., Mammalian Cell Line—Neutral Red Uptake); phototoxicity	OECD 432	USD 6 K	25
Skin sensitization (in vitro)	OECD 442 C/442 E	USD 10 K	60
Eye irritation/corrosion screen	OECD 496	1 K	20
**Ecotoxicity**			
Acute toxicity, bioluminescent bacteria (*Aliivibrio fischeri*), in vitro	ASTM STP766, in vitro assay	USD 7 K	20
Aquatic bioconcentration factor	* Estimated from experimentally measured K_OW_ (if organic)	NA	1–7
**BA3**			
**Chemical/Physical Characterization**			
Hydrolysis (rate) *	ASTM 895, OECD 111, EPA 712-C-08-012	USD 10 K	60 for all four
Photolysis (rate) *	ASTM E896, OECD 316, EPA 712-C-08-013	USD 10 K	
Persistence *	OECD 301, 310, 302 C, ASTM E1279, OPPTS 835.3180	USD 10 K	
Koc (Kd) *	ASTM E1195-01 (Withdrawn 2013, No Replacement), OECD 106 (recommended), OECD 121	USD 10 K	
**Human Health (specific exposure tests determined by professional judgment)**			
Acute oral toxicity	ASTM E1163, OECD 401, OECD 420, OECD 423, OECD 425, EPA 712-C-02-189, EPA 712-C-02-190	USD 13 K	74
Acute inhalation toxicity	OECD 403, OECD 436, EPA 712-C-98-193	USD 15 K	90
Acute dermal toxicity	OECD 402, EPA 712-C-98-192	USD 9 K	30
Skin irritation/corrosion	OECD 439, OECD 404, EPA 712-C-98-196	USD 7 K	30
Skin sensitization (3-pack in vitro)	OECD 442	USD 16 K	50
Additional in vitro genotoxicity tests (if reverse mutation results are positive):			
Genotoxicity, Chinese Hamster Ovary Test, in vitro	ASTM E1262, OECD 473	USD 21 K	65
Genotoxicity, Mouse Lymphoma Assay, in vitro	ASTM E1280, OECD 490	USD 21 K	56
Ecotoxicity *			
Aquatic toxicity—in vivo			
Acute aquatic organism toxicity *	ASTM E729, ASTM E1192, EPA-821-R-02-012	USD 25 K	60
Chronic aquatic organism toxicity *	EPA-821-R-02-013	USD 20	60
Aquatic plant (algae) toxicity *	OECD 201	USD 8	60
**BA4**			
**Chemical/Physical Characterization**			
Biodegradation (rate) *	ASTM E1279	USD 15	30
Leaching study *	OPPTS 835.1240	NA	
Treatability (select the test most relevant to manufacturing conditions and facility capabilities)			
Aerobic sewage treatment *	OECD 303, ASTM E1625	USD 15	30
Biodegradation in activated sludge *	OECD 311, ASTM E2170	USD 17	30
Biodegradation in wastewater *	OECD 314	USD 10	30
**Human Health (specific exposure tests determined by professional judgment)**			
28-day repeated dose, oral	OECD 407, EPA 712-C-00-366	USD 94 K	125
28- or 14-day repeated dose, inhalation	OECD 412	USD 180 K	120
Additional genotoxicity tests (if in vitro genotoxicity results are positive):			
Genotoxicity, in vivo (mouse micronucleus)	OECD 474	USD 17 K	65
Genotoxicity, Hepatic COMET Assay, in vivo	OECD 489	USD 15 K	65
Ecotoxicity *			
Bioconcentration and bioaccumulation *	ASTM E1676, OECD 317	varies	
Aquatic toxicity (chronic/sub-lethal) in vivo (three species) *			
Water flea (*Ceriodaphnia dubia*) (7 day) *	EPA-1002.2; ASTM E1295; ISO 20665	USD 50 K (all three)	30
Fathead Minnow (*Pimephales promelas*) (7 day) *	OECD 229		
Green algae (*Pseudokirchneriella subcapitata or Raphidocelis subcapitata*) *	OECD 201		
Freshwater Whole Effluent Aquatic Toxicity	EPA-821-R-02-013, EPA 821-B-00-004	USD 11–19	60
Terrestrial/soil invertebrate toxicity (chronic)		USD 80–130 K	90
Earthworm reproduction (*Eisenia fetida*/*Eisenia andrei*)—56 day *	ISO 11268-2; OECD 222	USD 70	90

Legend: ASTM = American Society for Testing and Materials; EPA = U.S. Environmental Protection Agency; ERDC = Engineer Research Development Center; ISO = International Organization for Standardization; NA = Not Applicable; OECD = Organization for Economic Co-operation and Development; OPPTS = EPA Office of Prevention, Pesticides, and Toxic Substances. * Needed only if expected to be released to the environment. ^i^ Costs averaged from an unofficial poll of contract research organizations and government laboratories from 2015–2016 and are expected to be dynamic.

## Data Availability

Not applicable.
